# Interactions of viruses in Cowpea: effects on growth and yield parameters

**DOI:** 10.1186/1743-422X-4-15

**Published:** 2007-02-08

**Authors:** KT Kareem, MA Taiwo

**Affiliations:** 1 Department of Microbiology University of Agriculture, Abeokuta, Nigeria; 2 Department of Botany and Microbiology University of Lagos, Akoka, Nigeria

## Abstract

The study was carried out to investigate the effects of inoculating three cowpea cultivars: "OLO II", "OLOYIN" and IT86D-719 with three unrelated viruses: *Cowpea aphid-borne mosaic virus *(CABMV), genus *Potyvirus*, C*owpea mottle virus *(CMeV), genus *Carmovirus *and *Southern bean mosaic virus *(SBMV), genus *Sobemovirus *singly and in mixture on growth and yield of cultivars at 10 and 30 days after planting (DAP). Generally, the growth and yield of the buffer inoculated control plants were significantly higher than those of the virus inoculated plants. Inoculation of plants at an early age of 10 DAP resulted in more severe effect than inoculations at a later stage of 30 DAP. The average values of plant height and number of leaves produced by plants inoculated 30 DAP were higher than those produced by plants inoculated 10 DAP. Most of the plants inoculated 10 DAP died and did not produce seeds. However, " OLOYIN" cultivar was most tolerant and produced reasonable yields when infected 30 DAP. The effect of single viruses on growth and yield of cultivars showed that CABMV caused more severe effects in IT86D-719, SBMV had the greatest effect on "OLO II" while CMeV induced the greatest effect on "OLOYIN". Yield was greatly reduced in double infections involving CABMV in combination with either CMeV or SBMV in "OLOYIN" and "OLO II", however, there was complete loss in yield of IT86D-719. Triple infection led to complete yield loss in all the three cultivars.

## Background

Cowpea is an annual tropical grain legume, which plays an important role in the nutrition of people in developing countries of the tropics and subtropics, especially in sub-Saharan Africa, Asia Central and South America. Due to its high protein content (20 – 25%), cowpea has been referred to as "poor man's meat". It is very palatable, highly nutritious and relatively free of metabolites or other toxins [1,2].

Cowpeas are susceptible to a wide range of pests and pathogens that attack the crop at all stages of growth [3]. These include insects, bacteria, fungi and viruses. Estimated losses due to virus infection have been variously put at between 10 and 100% [4,5], depending on the virus-host-vector relationships as well as the prevailing epidemiological factors. Host- plant resistance is currently the most effective method for the control of cowpea virus diseases in Africa. Thus, an adequate knowledge of the viruses and the strains occurring in the main cowpea-growing areas of Africa is a pre-requisite for effective control [6].

Out of more than 20 viruses reported on cowpea from different parts of the world [7-9] nine are known to infect the crop naturally in Nigeria [10-12]. They include the following viruses: *Cowpea aphid-borne mosaic virus *(CABMV), genus *Potyvirus*, *Cowpea yellow mosaic virus *(CPMV), genus *Comovirus*, *Southern bean mosaic virus *(SBMV), genus *Sobemovirus*, *Cowpea mottle virus *(CMeV), genus *Carmovirus*, *Cowpea golden mosaic virus *(CPGMV), genus *Bigeminivirus*, *Cucumber mosaic virus *(CMV), genus *Cucumovirus*, *Cowpea mild mottle virus *(CPMMV), genus *Carlavirus*, *Sunn-hemp mosaic virus *(SHMV), genus *Tobamovirus *and *Blackeye mosaic virus *(BICMV), genus *Potyvirus*. On the basis of geographical distribution, pathogenic variability and yield losses, CABMV, CPMV and occasionally SBMV are the most important viruses in Nigeria. CMeV, CPGMV and CMV are of localized importance while CPMMV and SHMV are not important. BICMV has a low rate of occurrence. Separation and identification of these viruses is by vector transmission, mechanical inoculation to diagnostic host species, symptomatology and serology [12].

Mixed viral infections in plant have been known for a long time and the mechanisms by which co-infecting viruses interact to alter host response have been a matter of speculation for a long time [13]. Mixed viral infections are common in plants and interactions may occur between viruses within the same host cell. Such viral interactions may be antagonistic or synergistic [14]. Antagonism usually occurs when the co-infecting viruses are related, resulting in interference [15] or cross-protection [16,17]. Synergism normally occurs in mixed infections when the pair of viruses involved are unrelated, resulting in more severe disease symptoms than those produced by single infections [18-20].

Surveys conducted by Shoyinka *et al*. [21] in Nigeria indicated that viruses occur in mixtures naturally and they cause mixed infections in cowpea crops. This research was designed to address the following objectives:

(a) to examine the effects of single and mixed infections by CABMV, CMeV and SBMV on growth and yield parameters of three Nigeria commercial cowpea cultivars and

(b) to determine the effect of age of plant at time of inoculation on the above parameters

## Materials and methods

One isolate each of *Cowpea Aphid-borne mosaic virus *(CABMV), genus *Potyvirus*, *Cowpea mottle virus *(CMeV), genus *Carmovirus *and *Southern bean mosaic virus *(SBMV) genus *Sobemovirus *were obtained from the International Institute of Tropical Agriculture (IITA), Ibadan, Nigeria. In order to increase the viruses and to ensure their availability, the three viruses were maintained by periodic transfer to healthy cowpea cultivar; Ife brown, in the greenhouse.

Three cowpea cultivars; IT86D-719, "OLO-II" and "OLOYIN" were used for the study. IT86D-719 obtained from IITA, Ibadan, "OLO-II" and "OLOYIN", which are two of Nigeria's commercial cowpea cultivars obtained from Mushin market, Mushin, Lagos State.

In the greenhouse, perforated plastic pots were filled with loamy soil obtained from Shodex Beautification Land Mark, Anthony, Lagos. The soil was sterilized with cypermethrin 10% E.C. to eliminate soil inhibiting microorganisms and enriched with farm yard manure. Two seeds of each cowpea cultivar were sown in each pot in October 2003. The plots were arranged in a split plot in a randomized complete block design with three replications. There were two factors, the virus treatment as main plot and cultivars as subplots. Each block consisted of 72 plastic pots and a total of 144 pots were utilized for the study. The seedlings were constantly watered and weeds removed manually as at when necessary. Inoculations were performed at 10 and 30 days after planting with single and mixed viruses consisting of CABMV, CMeV, SBMV, CABMV+CMeV, CABMV+SBMV, CMeV+SBMV and CABMV+CMeV+SBMV.

Virus extracts from infected plants maintained in greenhouse were prepared by grinding infected leaves with sterilized pestles and mortars in buffer. The buffer used throughout the study was 0.05 M dipotassium hydrogen orthophosphate (K_2_HPO_4_) pH 7.5 at a ratio of 1:2 (tissue weight: buffer volume). Leaf surfaces of plants to be inoculated were dusted with carborundum (180 grit) and pestles were used to apply the inoculum. Mixed virus inocula were prepared by weighing the leaves containing the different viruses separately and grinding equal weights of the infected leaves with sterilized pestles and mortars in K_2_HPO_4 _at a ratio of 1:2 wt/vol. The plants that served as control were inoculated with buffer solution only. Hands were thoroughly washed with detergent between treatments to prevent contamination. All the inoculated plants were rinsed with water after inoculation and kept in a greenhouse with temperatures at 25–28°C.

The effect of the different inocula on the growth parameters such as number of leaves and plant heights were determined by counting the number of leaves and measuring the heights of plants (in centimeters) that received the different treatments including controls and the averages of each parameter per replicate were determined. The effect of the different inocula on yield parameters was also determined by counting the number of flowers per plant, number of pods per plant length of each pod, and number of seeds per plant that received the different treatments including the controls replicate wise. A sensitive weighing balance (Mettler Toledo) was used to determine the weight of seeds after drying the seeds in the sun for seven days.

The statistical package for social scientist (SPSS) was used for the analysis of the data obatained. Duncan's multiple range test was used to determine the level of significance between the virus treatments at 5% probability level.

## Results

### Effect of virus inocula and age of plant at time of inoculation on growth and yield of "OLO II" cultivar

From the results in Table 1, all the treatments at 10 days after planting (DAP) had significantly different reduction in the heights of inoculated plants compared with buffer inoculated control plants. However, the heights of plants inoculated 30 DAP with CABMV alone and CMeV alone were not different from those of the controls. Inoculation of the "OLO II" with a mixture of the three viruses resulted in the greatest reduction in plant height with the average values at 12.7 cm and 22.1 cm for plants inoculated at 10 and 30 DAP compared with the 69.3 cm for the buffer inoculated plants. Infections with SBMV either singly or in combination with CMeV resulted in the greatest reduction in plant heights.

**Table 1:** The Effects of Virus Inoculum and Stage of Plant Growth at the Time of Inoculation on Growth and Yield of Cowpea Cultivar "OLO II"

Inoculum	API	Plant Height(cm)	No. of Leaves	No. of Flowers	No. of Pods	Pod length(cm)	Seed no./pod	Seed Weight(g)
CABMV	10	20.8bc	12.0b	0.0*	0.0	0.0	0.0	0.0
	30	66.6a	23.0ab	4.7a	1.3b	4.1b	2.0b	0.05b
								
CMeV	10	26.7bc	7.0c	0.0	0.0	0.0	0.0	0.0
	30	66.5a	18.1b	0.7b	0.0	0.0	0.0	0.0
								
SBMV	10	19.1c	3.5c	0.0	0.0	0.0	0.0	0.0
	30	50.6ab	15.8b	1.0b	0.0	0.0	0.0	0.0
								
CABMV+	10	22.4bc	2.9c	0.0	0.0	0.0	0.0	0.0
CMeV	30	32.8b	11.0b	0.7b	0.7b	2.1b	1.3b	0.04c
								
CABMV+	10	25.7bc	3.1c	0.0	0.0	0.0	0.0	0.0
SBMV	30	36.3b	14.9b	0.7b	0.0	0.0	0.0	0.0
								
CMeV+	10	13.5c	3.6c	0.0	0.0	0.0	0.0	0.0
SBMV	30	56.9ab	25.1ab	5.0a	2.0a	8.1a	3.7a	0.1b
								
CABMV+	10	12.7c	2.4c	0.0	0.0	0.0	0.0	0.0
CMeV+	30	22.1bc	10.2b	0.0	0.0	0.0	0.0	0.0
								
SBMV	10	65.5a	18.7b	3.7a	2.7a	9.4a	3.3a	0.14b
BUFFER	30	69.3a	40.4a	4.7a	3.0a	10.4a	4.3a	0.16a

API-Age of plant at inoculation; CABMV-*Cowpea aphid-borne mosaic virus*, CMeV-*Cowpea mottle virus*, SBMV-*Southern bean mosaic virus*.

Each value is the mean of 3 replicates. In each column means followed by the same letter are not significantly different (P < 0.05) according to Duncan's multiple range test.

*All zero (0) values carry the letter c according to Duncan's multiple range test.

Similarly, all plants inoculated at 10 and 30 DAP produced significantly fewer leaves compared with the buffer inoculated control except for inoculation with CABMV alone (Table 1). The number of leaves was greatly reduced when the plants were inoculated with a mixture of the three viruses with average values of 2.4 and 10.2 for plants inoculated at 10 and 30 DAP respectively. These values were significantly different from the 18.7 and 40.4 observed for the buffer inoculated control plant of the same age.

Still from Table 1, virus treated plants produced lower yields compared with the control. The effect of the viruses on the yield of "OLO II" revealed that plants inoculated with single and mixed viruses at 10 DAP produced no flowers or flowers aborted prematurely and therefore no seeds were produced. However, plants inoculated with CABMV, CABMV+CMeV and CMeV+SBMV at 30 DAP produced a few seeds.

### Effect of virus inocula and age of palnt at time of inoculation on growth and yield of IT86D-719 cultivar

In table 2, severe stunting was induced in plants inoculated with a mixture of the three viruses. Inoculations with CABMV alone and in mixture with SBMV at 10 (DAP) resulted in the greatest reduction in plant height. Single inoculations with CMeV, SBMV or double inoculation with CABMV+ CMeV and CABMV+SBMV resulted in fairly similar effects on plant height. Inoculation of cowpea cultivars IT86D-719 with the three viruses caused the greatest reduction in number of leaves at 10 DAP with an average value of 0.6 while CABMV alone and its combination with SBMV also resulted in significantly reduced number of leaves with 1.0 and 3.3 respectively compared to 9.0 for the control plant at the same age of inoculation. The effect of inoculating with CMeV alone and in combination with SBMV were not significantly different from those of the controls in the number of leaves at 10 and 30 DAP.

**Table 2:** The Effect of Virus Inoculum And Stage of Plant Growth at the Time of Inoculation on Growth and Yield of Cowpea Cultivar IT86D-719

INOCULUM	API	Plant height(cm)	No. of leaves	No. of Flowers	No. of Pods	Pod length(cm)	Seed no./pod	Seed Weight(g)
CABMV	10	21.8b	1.0c	0.0*	0.0	0.0	0.0	0.0
	30	40.4a	10.0b	1.0b	0.0	0.0	0.0	0.0
								
CMeV	10	25.9b	5.2b	0.0	0.0	0.0	0.0	0.0
	30	37.9ab	16.0a	3.0ab	0.0	0.0	0.0	0.0
								
SBMV	10	28.5b	14.0a	0.0	0.0	0.0	0.0	0.0
	30	38.7ab	14.1a	6.3a	3.3ab	9.1a	6.7a	0.1b
								
CABMV+	10	33.5ab	14.2a	0.3bc	0.0	0.0	0.0	0.0
CMeV	30	37.8ab	6.0b	0.7bc	0.0	0.0	0.0	0.0
								
CABMV+	10	24.9b	3.3c	0.0	0.0	0.0	0.0	0.0
SBMV	30	36.1ab	6.9b	0.0	0.0	0.0	0.0	0.0
								
CMeV+	10	25.2b	8.8b	1.7b	0.0	0.0	0.0	0.0
SBMV	30	41.7a	24.6a	1.7b	0.0	0.0	0.0	0.0
								
CABMV+	10	19.8c	0.6c	0.0	0.0	0.0	0.0	0.0
CMeV+	30	25.7b	12.6a	0.0	0.0	0.0	0.0	0.0
								
SBMV	10	37.6ab	9.0b	4.3a	2.7b	7.9b	5.0b	0.2a
BUFFER	30	42.3a	13.0a	6.7a	4.0a	8.6a	5.0b	0.2a

API-Age of plant at inoculation; CABMV-*Cowpea aphid-borne mosaic virus*, CMeV-*Cowpea mottle virus*, SBMV-*Southern bean mosaic virus*.

Each value is the mean of 3 replicates. In each column means followed by the same letter are not significantly different (P < 0.05) according to Duncan's multiple range test.

*All zero (0) values carry the letter c according to Duncan's multiple range test.

In the same Table 2, the various virus treatments resulted in a complete loss in yield of cultivar IT86D-719 except for plants inoculated with SBMV at 30 DAP. A few flowers were produced by some of the plants but seeds were generally not produced.

### Effect of virus inocula and age of plant at time of inoculation on growth and yield of "OLOYIN" cultivar

From the result in table 3, the effect of CMeV+SBMV and a combination of the three viruses produced the most severe effect on plant height with average values of 7.8 cm to 8.1 cm respectively. In single viral infections, the severity of infection on plant height was milder than those of the mixed infections with CMeV having the least effect at early and late inoculations with mean values of 23.1 cm and 27.7 cm respectively. The effect of SBMV on plant height was not significantly different from the controls at early inoculation. The greatest reduction in number of leaves was observed in plants inoculated with a mixture of CABMV and CMeV at 10 and 30 DAP with means of 5.7 and 13.6 compared with 18.9 and 25.0 for buffer inoculatd control respectively. In single infections, CMeV produced the greatest effect on number of leaves followed by CABMV while SBMV induced the least effect. However, in double infections, it was the mixture of CABMV and CMeV that produced the greatest effect (Table 3).

**Table 3:** The Effects of Virus Inoculum and Stage of Plant Growth at the Time of Inoculation on Growth and Yield of Cowpea Cultivar "OLOYIN" Cultivar

INOCULUM	API	Plant height(cm)	No. of Leaves	No. of Flowers	No. of Pods	Pod length(cm)	Seed no./pod	Seed Weight(g)
CABMV	10	24.9b	11.1b	0.0*	0.0	0.0	0.0	0.0
	30	33.8ab	20.9b	3.7b	3.0b	9.6a	6.7a	0.09b
								
CMeV	10	23.1b	6.9c	0.0	0.0	0.0	0.0	0.0
	30	27.7b	17.1b	3.3b	2.0b	7.1b	2.0b	0.01c
								
SBMV	10	28.2b	17.2b	0.0	0.0	0.0	0.0	0.0
	30	40.4a	18.0b	2.3b	1.3b	10.2a	6.7a	0.1b
								
CABMV+	10	11.9c	5.7c	0.0	0.0	0.0	0.0	0.0
CMeV	30	26.7b	13.6b	1.3b	1.0b	5.2b	3.3ab	0.06b
								
CABMV+	10	12.4c	17.9b	0.0	0.0	0.0	0.0	0.0
SBMV	30	30.4b	22.1a	2.0b	1.7b	4.1b	2.7b	0.05b
								
CMeV+	10	7.8c	12.8b	0.0	0.0	0.0	0.0	0.0
SBMV	30	23.8b	23.4a	3.0b	2.0b	4.0b	1.7b	0.09b
								
CABMV+	10	8.1c	9.3c	0.0	0.0	0.0	0.0	0.0
C MeV+	30	12.2c	14.4b	1.0b	0.0	0.0	0.0	0.0
								
SBMV	10	29.7b	18.9b	6.0a	5.3a	9.3a	4.3a	0.145a
BUFFER	30	42.7a	25.0a	7.0a	6.3a	10.0a	5.3a	0.145a

API-Age of plant at inoculation; CABMV-*Cowpea aphid-borne mosaic virus*, CMeV-*Cowpea mottle virus*, SBMV-*Southern bean mosaic virus*.

Each value is the mean of 3 replicates. In each column means followed by the same letter are not significantly different (P < 0.05) according to Duncan's multiple range test.

*All zero (0) values carry the letter c according to Duncan's multiple range test.

Still in Table 3, no flowers or seeds/pods were produced by all the plants inoculated 10 DAP. This was significantly different from the controls which had an average value of 6.0 flowers and 4.3 seeds/pods. However, the plants inoculated at 30 DAP produced flowers and seeds except in plants inoculated with combination of the three viruses, which produced no seeds (Table 3).

## Discussion

The results of this study indicate that the three cowpea cultivars used in this investigation were susceptible to the three viruses and the viruses replicate in them in both single and mixed infections. There have been reports of *Cowpea aphid-borne mosaic virus *[22,23]*, Cowpea mottle virus *[24] and *Southern bean mosaic virus *[21] existing in Nigeria.

Early infection of cowpea cultivars with single or mixed viruses resulted in shortening of internode, apical necrosis, which led to cessation of growth, stunting and eventual plant death. This fact is substantiated by the research of Pio-Ribeiro *et al*. [25] which indicated that CABMV and Cucumber mosaic cucumovirus interact synergistically to produce cowpea stunt, a disease characterized by severe stunting and yields loss. Also, Niblett and Claflin [26] and Uyemoto *et al*. [27] demonstrated that maize dwarf mosaic virus (MCMV) in maize (*Zea mays*) to induce the necrosis disease which resulted in up to 91% yield loss and death of many plants especially when infection occurred early. This is also confirmed by this study where early infection of cultivars by viruses resulted in more drastic response than infection at a later stage of growth.

The results of the effect of single and mixed virus infection on the growth parameters of cowpea cultivars showed significant reduction in the growth parameters in plants inoculated at an early age. Inoculation of cultivars with CABMV, CMeV and SBMV alone and in mixed infection at 10 days after planting resulted in fewer leaves and reduced plant height than inoculation at 30 days after planting. This finding is in agreement with the previous report that the younger the plants at the time of viral infection the greater the severity of disease symptoms [28].

The result of this study showed that single or multiple inoculation of the three cultivars with CABMV, CMeV and SBMV at an early age (10 days after planting) resulted in complete loss in yield. The earlier a virus infects a plant, the more severe the reduction in yield [29,30]. Gilmer *et al*. [31] showed that inoculation of cowpea cultivars with Cowpea yellow mosaic virus (CpMV) 7 days after emergence reduced yield by 40%-60% compared to the 10%-15% loss in yield when plants were inoculated at flowering. In "OLOYIN" cultivar yields were not affected when plants were infected 30 days after planting but in "OLO II" and IT86D-719, there was complete loss in yield in almost all the treatments irrespective of time of inoculation. This is in agreement with the report of Wells and Deba [32] which reported up to 100% yield loss in cowpea in Western Nigeria due to CpMV. Raheji and Leleji [5] also reported that CABMV resulted in a complete loss of an irrigated cowpea crop in Northern Nigeria. Owolabi *et al*. [33] reported that infection of some Nigeria commercial cowpea lines by BICMV could result in a complete loss in yield.

The losses recorded in the commercial variety vary from one cultivar to another. "OLO II" is more susceptible to viral infection than "OLOYIN". This in accordance with the report of Owolabi et al. [33] that when both Ife brown and the Nigerian B7 cultivars were each single inoculated with BICMV and CPMV, the effect was more pronounced on the Nigerian B7 than the Ife brown. A report says that many of the severe disease caused by viral synergistic interactions occur when one of the infecting viruses is a member of the genus Potyvirus [34]. This statement is substantiated by the result obtained for the yield of the three cultivars where the mixture of CABMV with either CMeV and or SBMV produced severe loss in yield. This may be due to virus infection depending among other factors on virus isolate, the presence of another virus, the tolerance of the infected cowpea cultivars and the stage of the host plant at the time of the infection [35-37].

The two commercial varieties used in this study are susceptible to the three viruses. Taiwo [12] reported that in Nigeria, the most economical, practicable and effective method of control of legume viruses is through the use of resistant varieties. Cowpea lines with individual or combined resistance to severe cowpea viruses has been identified at IITA [11]. Source of resistance have also been identified in soybean [38]. Such legume lines are being tested in different localities for selection of the best locally adapted varieties with multiple virus resistance [11]. However, the rate of acceptance and utilization of such resistant varieties is rather poor.

There is need to prevent early infection and complete loss of crop by producing virus free seeds and controlling virus vectors. There is also a need to ensure availability of acceptable horticulturally desirable cowpea cultivars with a high level of resistance to cowpea viruses for the nation to sustain its high level of productivity.
